# Estradiol Reshapes Cell-Type-Dependent Basal Redox Set-Points in Colorectal Carcinoma Cells

**DOI:** 10.3390/biomedicines14071577

**Published:** 2026-07-14

**Authors:** Natasa Z. Djordjevic, Nemanja Vučićević, Milica Pešić

**Affiliations:** 1 Department of Natural and Mathematical Sciences, State University of Novi Pazar, Vuka Karadzica 9, 36300 Novi Pazar, Serbia; 2 Department of Mathematics and Informatics, Faculty of Science, University of Kragujevac, Radoja Domanovića 12, 34000 Kragujevac, Serbia; nemanja.vucicevic@pmf.kg.ac.rs; 3 Department of Neurobiology, Institute for Biological Research “Siniša Stanković”—National Institute of the Republic of Serbia, University of Belgrade, Despota Stefana 142, 11108 Belgrade, Serbia

**Keywords:** estradiol, colorectal cancer, oxidative stress, redox homeostasis, mathematical analysis

## Abstract

**Background/Objectives**: Since redox balance is critical to colorectal cancer cell survival, and estradiol, a potent antioxidant, correlates with reduced disease incidence, understanding the redox basis of cellular responsiveness to estradiol is essential for advancing therapeutic insight. This study evaluates the adaptive and maladaptive redox responses of colorectal carcinoma cells to estradiol treatment by defining the basal redox set-point as the intracellular balance between pro-oxidants and antioxidants. **Methods**: Human colorectal cancer cell lines HCT-116 and SW-480 were treated for 24 h with pregnancy-range and pharmacological-range concentrations of estradiol. Redox biomarkers (superoxide anion/O_2_^.−^, hydrogen peroxide/H_2_O_2_, nitric oxide/NO, reduced glutathione/GSH and oxidized glutathione/GSSG), cell viability, and basal migration were analyzed. Correlation, network topology, PCA, and Jaccard similarity analyses were applied to characterize basal redox set-points and quantify estradiol-induced changes in redox profiles in the two cell lines. **Results**: HCT-116 cells exhibited an O_2_^.−^-centered redox set-point associated with NO and GSH. SW-480 cells displayed a H_2_O_2_-centered redox set-point associated with NO and GSH. In HCT-116 cells, estradiol triggered a maladaptive response associated with antioxidant activation and reduced proliferation. Conversely, SW-480 cells exhibited an adaptive response characterized by modulation of NO levels and the GSH pool and associated with increased proliferation. **Conclusions**: These findings identify redox set-point organization as a potential determinant of estradiol responsiveness in colorectal cancer cells. From a clinical perspective, characterizing basal redox set-points in patient-derived tumor cells may enable stratification of colorectal cancer patients by predicted responsiveness to redox-modulating therapies, informing personalized treatment.

## 1. Introduction

According to the World Health Organization (WHO), colorectal cancer accounts for approximately 10% of all diagnosed cancers, with around 1.93 million new cases worldwide each year, making it the third most commonly diagnosed cancer [1]. It is the second leading cause of cancer-related mortality globally, with approximately 916,000 deaths annually, corresponding to a mortality-to-incidence ratio of 46.47%. Epidemiological studies demonstrate a lower incidence of colorectal cancer in women, especially in premenopausal women, than in men of the same age [2,3,4]. In addition, numerous studies indicate that multiple pregnancies and postmenopausal replacement therapy reduce the risk of colorectal cancer in women [5,6,7,8]. These studies suggest that estradiol has a protective effect and may reduce the risk of colorectal cancer in women. This is supported by evidence from animal models and in vitro studies, which demonstrate estradiol-mediated protection against colorectal carcinogenesis [9,10,11,12]. The mechanisms by which estradiol may reduce the risk of colorectal cancer are not yet fully elucidated.

One of the proposed mechanisms by which estradiol may exert protective effects against colorectal cancer involves the modulation of cellular redox balance. In contrast to normal cells, colorectal cancer cells exhibit elevated reactive oxygen species (ROS) production, primarily arising from mitochondrial dysfunction and hyperactivation of NADPH oxidases, particularly NOX1 [13,14]. Cytosolic enzymes, lysosomes, peroxisomes, and the endoplasmic reticulum represent additional intracellular sources of ROS in colorectal cancer cells [15]. ROS, including the superoxide anion radical (O_2_^.−^) and its dismutation product hydrogen peroxide (H_2_O_2_), act as critical signaling molecules in cancer. They regulate carcinogenesis, cancer cell survival and proliferation, metabolic reprogramming, angiogenesis, and metastatic progression [16]. However, in addition to their procarcinogenic role, ROS can also exert anticancer effects, depending on their intracellular concentration. When produced at high levels, ROS can induce cancer cell death through multiple regulated cell death pathways, including apoptosis, necroptosis, pyroptosis, ferroptosis, and autophagy. To counteract the destructive potential of excessive ROS, cancer cells enhance their antioxidative defense networks, maintaining redox homeostasis and supporting survival and proliferation [16].

Similarly, nitric oxide (NO) serves as a critical redox and signaling mediator, with a complex and context-dependent role in cancer cells. Its procarcinogenic or anticancer effects depend on NO’s cellular source (tumor cell-derived versus exogenous), the tumor stage (early versus metastatic), and the tumor type (solid versus hematological) [17]. Produced by cells in the tumor microenvironment, NO exerts concentration-dependent effects: at low levels it promotes tumor growth, whereas at high levels it induces cancer cell death [17]. Conversely, within cancer cells, elevated NO production suppresses tumor growth and metastasis, while low intracellular NO levels can trigger apoptosis [18]. Within cancer cells, inducible nitric oxide synthase (iNOS) is a major source of sustained NO production and contributes to the regulation of redox homeostasis, proliferation, migration, apoptosis, and cellular stress responses [17,19]. Increased iNOS expression and NO production have been reported in colorectal cancer and associated with cancer cell survival, aggressiveness, and resistance to conventional therapies [20,21].

Given the critical role of redox balance in colorectal cancer cell survival and progression, targeting redox balance represents a potential therapeutic approach. Estradiol is the most potent female sex steroid hormone and its role in enhancing antioxidative defense is well documented [22]. Estradiol exerts its antioxidative effects in a receptor-independent manner as a direct scavenger of free radicals and via membrane-associated estrogen receptors (G protein–coupled estrogen receptor—GPER) and nuclear estrogen receptors (ERα and ERβ) [22,23,24].

As a liposoluble steroid hormone, estradiol easily passes through the cell membrane. Due to its ability to release hydrogen from its phenol-hydroxyl ring A, it acts as a strong chain breaking antioxidant, which can stop the chain reaction of lipid peroxidation [22]. Studies by Arteaga et al. show that the antioxidative potential of estradiol is 10 to 100 times higher than vitamin E, making it a very effective antioxidant [25].

By acting through GPER, estradiol is able to rapidly activate the PI3K/Akt/GSK-3β and ERK1/2 signaling pathways within seconds or minutes, which are involved in enhancing antioxidative defenses, suppressing inflammatory responses, and inhibiting apoptosis [23]. In colorectal cancer cells, GPER exerts tumor-suppressive effects primarily through modulation of redox-sensitive signaling pathways. Specifically, GPER activation triggers a controlled increase in intracellular ROS, leading to activation of ERK1/2 signaling and concomitant inhibition of NF-κB activity. This redox-dependent signaling cascade results in G2/M cell cycle arrest, suppression of cell proliferation, induction of mitochondrial-dependent apoptosis, and activation of endoplasmic reticulum stress. GPER expression is significantly reduced in human colorectal cancer tissues compared with normal intestinal epithelium. Clinically, low GPER expression in tumor tissue is associated with poorer prognosis, whereas higher GPER expression correlates with improved survival, particularly in women with advanced-stage (III and IV) colorectal cancer [26,27,28].

ERα and ERβ are nuclear estrogen receptors that, upon binding to estradiol, function as ligand-activated transcription factors regulating gene expression involved in cell survival, redox homeostasis, and inflammation [24,29,30]. Despite their structural similarity, ERα and ERβ exert largely opposing biological effects. ERα predominantly induces proliferative and anti-apoptotic gene programs, whereas ERβ activates antiproliferative and pro-apoptotic signaling pathways [24]. Through ERα, estradiol upregulates the expression of key antioxidant enzymes, including glutathione peroxidase (GPx), catalase (CAT), superoxide dismutase (SOD), and glutathione S-transferase (GST), thereby enhancing cellular antioxidative capacity and redox homeostasis [29]. In addition, ERα suppresses iNOS expression, contributing to reduced nitrosative stress [30]. In the intestinal epithelium, ERβ is the predominant estrogen receptor, promoting epithelial integrity and stimulating mucosal repair through autophagy [31,32]. In contrast, ERα contributes to epithelial regeneration and ion transport but may exacerbate intestinal inflammation through activation of the NLRP3 inflammasome, underscoring their distinct and often antagonistic roles in intestinal homeostasis [32,33]. ERβ expression is markedly reduced in colorectal cancer tissues compared with normal intestinal epithelium, whereas ERα expression is significantly increased in colorectal carcinoma cells [31,32,33]. Experimental and clinical evidence indicates that the cancer-preventive effects of estradiol correlate with increased ERβ and decreased ERα expression in target tissues [31,34]. Clinically, low ERβ expression is associated with poorer survival and higher recurrence rates, whereas elevated ERα levels correlate with liver metastasis and unfavorable prognosis [31].

Although epidemiological and experimental evidence suggests that estradiol may protect against colorectal cancer, and its direct and indirect antioxidative effects are well documented, it remains unclear how estradiol affects redox homeostasis and functional responses in a cell line- and dose-dependent manner.

The aim of this study was to characterize redox homeostasis in colorectal cancer cell lines HCT-116 and SW-480 and to investigate their adaptive and maladaptive redox responses to estradiol, a potential therapeutic agent in human colorectal cancer.

To gain a systems-level perspective on redox homeostasis, we applied network-based and multivariate analytical approaches. Redox biomarkers were modeled as nodes in a correlation network, where edges represent statistically significant Pearson correlations (*p* ≤ 0.05). Network topology was characterized by the number of edges, network density, and degree centrality of individual biomarkers. To quantify global shifts in the redox phenotype induced by estradiol, Principal Component Analysis (PCA) was performed on standardized biomarker data, and Euclidean distances from the basal state were calculated both in the full five-dimensional biomarker space and in the PC1–PC2 plane. Structural similarity between control and estradiol-treated networks was assessed using the Jaccard similarity index.

## 2. Materials and Methods

### 2.1. Reagents and Chemicals

All reagents used in this study were analytically graded and obtained from Merck (Sigma-Aldrich, Darmstadt, Germany) and Gibco (Grand Island, NY, USA). Absorbance measurements were performed using a microplate reader (RT-6100, Rayto Life and Analytical Sciences Co., Ltd., Shenzhen, China). Statistical analyses were performed using IBM SPSS Statistics for Windows, version 27.0 (IBM Corp., Armonk, NY, USA).

### 2.2. Cell Culture Maintenance

Human colorectal cancer cell lines HCT-116 and SW-480 were obtained from the American Type Culture Collection (ATCC, Manassas, VA, USA). Prior to the experiments, the cells were cultured at 37 °C in a humidified atmosphere containing 5% CO_2_ in 75 cm^2^ tissue culture flasks. Dulbecco’s Modified Eagle Medium (DMEM) supplemented with 10% fetal bovine serum and 1% antibiotic/antimycotic solution containing 10,000 units/mL penicillin, 10 mg/mL streptomycin, and 25 μg/mL amphotericin B was used as the culture medium.

### 2.3. Treatments

At approximately 90% confluence, cells were harvested and 100.000 cells per well were seeded into 96-well plates at a volume of 0.1 mL per well for the MTT assay. After 24 h of incubation, cells in both 96-well plates and culture T75 flasks were treated with different concentrations of estradiol. A 10 mM stock solution of estradiol was prepared by dissolving the compound in absolute ethanol, and subsequently serially diluted in DMEM to obtain the final working concentrations used for cell treatment. The concentrations of 10^−8^ and 10^−7^ mol/L correspond to the lower and upper physiological ranges observed during pregnancy, respectively, whereas 10^−6^ and 10^−5^ mol/L represent supraphysiological, pharmacological doses. Both the 96-well plates and culture flasks containing treated and untreated cells (control) were incubated at 37 °C in a humidified atmosphere with 5% CO_2_ for 24 h. The control and each treatment condition were tested in quadruplicate in three independent experiments.

### 2.4. MTT Assay

The effects of estradiol on cell viability were determined using the MTT assay, which is based on the reduction of the yellow tetrazolium salt MTT (3-[4,5-dimethylthiazol-2-yl]-2,5-diphenyltetrazolium bromide) by mitochondrial dehydrogenases in living cells to purple formazan [35]. After treatment, cells were washed with phosphate-buffered saline (PBS), and 100 μL of MTT solution (5 mg/mL) was added to each well. The cells were incubated at 37 °C in a humidified atmosphere containing 5% CO_2_ for 3 h. Following incubation, the MTT solution was removed, and the formed purple formazan was dissolved by adding 150 μL of dimethyl sulfoxide (DMSO) to each well. Absorbance was measured using a microplate reader (RT-6100, Rayto Life and Analytical Sciences Co., Ltd., Shenzhen, China) at 550 nm, and cell viability was calculated as (absorbance of treated cells/absorbance of control cells) × 100 and expressed as %.

### 2.5. Determination of Oxidative–Antioxidative Components

After treatment, cells from T75 flasks were harvested with trypsin, centrifuged at 2500 rpm for 5 min, and washed twice with PBS. The washed cells were resuspended in PBS and adjusted to a concentration of 10^6^ cells/mL for subsequent analyses.

The concentration of O_2_^.−^ in the cells was determined using the nitroblue tetrazolium (NBT) assay, which is based on the reduction of NBT in the presence of O_2_^.−^ [36]. A total of 100 μL of cell suspension was added per well in a 96-well plate and mixed with 0.1% NBT. After incubation for 45 min at 37 °C in a humidified atmosphere containing 5% CO_2_, the concentration of O_2_^.−^ was measured using a microplate reader at 630 nm and expressed as nmol/10^6^ cells.

To determine the concentrations of H_2_O_2_ and nitrites (NO_2_^−^), used as an indicator of NO concentration, cell membranes were disrupted by three cycles of freezing at −80 °C and thawing at 37 °C for 15 min each, followed by centrifugation for 20 min at 4500 rpm. The concentrations of H_2_O_2_ and NO_2_^−^ were measured in the supernatant.

The concentration of H_2_O_2_ was determined based on the oxidation of phenol red in the presence of horseradish peroxidase (HRP, type II) as a catalyst [37]. A total of 50 μL of supernatant was mixed with 50 μL of reaction mixture containing 0.28 mM phenol red and HRP (1 U/mL). After incubation for 1 h at 37 °C in a humidified atmosphere containing 5% CO_2_, the reaction was stopped by adding 1 N NaOH, and absorbance was measured using a microplate reader at 600 nm. H_2_O_2_ concentrations were calculated from a H_2_O_2_ standard curve (2.5–20 nmol/mL) and expressed as nmol/10^6^ cells.

The concentration of NO_2_^−^, as an indicator of NO levels, was determined using the Griess method [38]. A total of 50 μL of cell supernatant was added per well in a 96-well plate, followed by 50 μL of sulfanilamide reagent (1% sulfanilamide in 5% phosphoric acid). After 10 min incubation at room temperature, 50 μL of 0.1% N-(1-naphthyl)ethylenediamine dihydrochloride (NED) was added, and the plate was incubated for an additional 10 min in the dark at room temperature. Absorbance was measured using a microplate reader at 550 nm, and NO_2_^−^ concentrations were calculated from a standard curve of NaNO_2_ (1.5–100 nmol/mL). Data are expressed as NO concentration in nmol/10^6^ cells.

Concentrations of reduced (GSH) and oxidized (GSSG) glutathione were determined after an extraction using the following protocol: control and treated cells pellet were resuspended in 2.25% sulfosalicylic acid, and they were lysed by intermittent freezing at −80 °C and thawing at 37 °C in 3 cycles for 15 min each, followed by a centrifugation for 20 min at 4500 rpm. Immediately after extraction of the cell supernatant, 4-vinylpyridine was added to the sample used for GSSG determination to prevent the spontaneous oxidation of GSH to GSSG.

In the supernatant, the concentration of GSH was determined based on GSH oxidation by 5.5-dithio-bis-6.2-nitrobenzoic acid (DTNB) [39]. A total of 20 μL of cell supernatant was added per well in a 96-well plate, followed by 160 µL 0.3 M disodium phosphate (Na_2_HPO_4_) and 20 µL 0.04% DTNB in 1% Na-citrate and incubated 10 min at room temperature. After incubation, absorbance was measured using a microplate reader at 405 nm. GSH concentrations were calculated from a GSH standard curve (1.5–100 nmol/mL) and expressed in nmol/10^6^ cells.

The concentration of GSSG was determined enzymatically using glutathione reductase (GR) [40]. A total of 50 μL of cell supernatant was added per well in a 96-well plate, followed by 100 μL of reaction mixture containing GR (1.0 U/mL), 0.2 mM NADPH, and 1.5 mM DTNB (10X) in 5% sodium bicarbonate (NaHCO_3_) and 80 μM potassium phosphate (K-PO_4_) buffer, pH 7.4. The plate was incubated for 10 min at room temperature, and absorbance was measured at 405 nm using a microplate reader. GSSG concentrations were calculated from a GSSG standard curve (1.5–50 nmol/mL) and expressed as nmol/10^6^ cells.

### 2.6. Cell Migration Assay

To assess the effect of estradiol on basal cell migration, only the estradiol concentration that did not cause more than a 5% change in cell viability was used.

Basal migration of HCT-116 cells was evaluated using a transwell migration assay after 24 h of treatment. Subconfluent cells were trypsinized and centrifuged at 800 rpm for 3 min. Cells were then resuspended at a concentration of 2 × 10^5^ cells/mL: untreated cells in serum-free medium, and treated cells in serum-free medium containing 10^−8^ mol/L estradiol. A total of 100,000 cells in 500 μL of suspension were added to the upper chamber of a transwell insert placed in a 24-well plate. For untreated cells, the lower chambers contained 750 μL medium with serum (positive control) and 750 μL serum-free medium (negative control). For treated cells, lower chambers contained serum-free medium containing estradiol. Control wells confirmed baseline migratory activity: medium with serum served as a positive control representing maximal migration, while serum-free medium served as a negative control representing minimal spontaneous migration. Estradiol effects were assessed relative to these baseline control, without establishing a chemotactic gradient. Plates were incubated for 6 h at 37 °C in a humidified atmosphere with 5% CO_2_.

After incubation, transwell inserts were washed with PBS, and cells on the lower side of the membrane were fixed with 4% paraformaldehyde for 20 min at room temperature, followed by two PBS washes. Cells remaining on the upper surface of the membrane were removed with a cotton swab. Migrated cells on the lower surface were stained with 0.1% Crystal Violet in 200 mM MES buffer (pH 6.0) for 10 min at room temperature, washed twice with distilled water, and air-dried. Membranes were carefully cut and placed into 100 μL of 10% acetic acid in a 96-well plate, and 80 μL of the solution was transferred to a new 96-well plate for absorbance measurement at 595 nm using a microplate reader. The percentage of migration was calculated as the ratio of the absorbance of each sample or negative control to that of the positive control, multiplied by 100.

### 2.7. Statistical Analysis

Data analyses were performed using SPSS for Windows (version 27.0). All measurements were conducted in quadruplicate in three independent experiments, and results are presented as mean ± SEM. Differences between untreated (control) and estradiol-treated cells were assessed using the independent-samples *t*-test. Prior to analysis, data normality was evaluated using the Shapiro–Wilk test. Statistical significance was accepted at *p* ≤ 0.05.

Pearson correlation coefficient (*r*) was used to assess relationships among redox biomarkers with statistical significance set at *p* ≤ 0.05, *p* ≤ 0.01, and *p* ≤ 0.001. Pearson correlation coefficients were calculated for all measured redox parameters and are presented in the Supplementary Data. Statistically significant correlations were visualized as network graphs, where nodes represent individual biomarkers and edges indicate significant correlations.

### 2.8. Mathematical and Network Analysis

Redox profile vector and data standardization. The redox profile of each cell line and treatment condition was represented as a five-dimensional vector x = (O_2_^.−^, H_2_O_2_, NO, GSH, GSSG). Prior to PCA and distance calculations, all biomarkers were standardized column-wise to z-scores: $z_{ij}\;=\;(x_{ij}\;-\;mean_{j})/sd_{j}$. This ensured comparability across biomarkers regardless of absolute scale.

Principal Component Analysis (PCA). PCA was applied to the standardized data matrix Z by computing the correlation matrix $R\;=\frac{1}{n\;-\;1}\cdot Z^{T}Z$ and solving the eigenvalue problem $Rv_{k}\;=\;\lambda_{k}\;v_{k}$. The percentage of explained variance for each component was calculated as $Explained\;variance_{k}\;=\frac{\lambda_{k}}{\sum_{j}\lambda j\;}\times 100$. PCA was used as an exploratory descriptive method; results are interpreted as descriptive patterns, not statistical hypothesis tests.

Distance from basal state. For each estradiol-treated condition, two distances from the corresponding untreated control were calculated: (1) the Euclidean distance in the full standardized five-dimensional biomarker space ($d_{5D}\;=\;∥z_{treated}\;-\;z_{control}∥$) and (2) the distance in the PC1 − PC2 plane:

$$d_{PC}\;=\sqrt{{\left(PC1_{treated}\;\;-\;PC1_{control}\right)}^{2}\;+\;{\left(PC2_{treated}\;-\;PC2_{control}\right)}^{2})\;}.$$

Correlation network analysis. Biomarkers were modeled as network nodes. An edge between two biomarkers was defined as a statistically significant Pearson correlation (*p* ≤ 0.05). The maximum number of possible undirected edges among 5 nodes is 10. Network density was calculated as D = m/10, where m is the number of significant edges. Normalized degree centrality for each biomarker *i* was computed as $C_{D}(i)\;=\;k_{i}/(n\;-\;1)\;=\;ki/4$, where *ki* is the number of significant edges connected to node *i*.

Jaccard similarity index. To quantify the structural similarity between estradiol-treated and control correlation networks, the Jaccard index was calculated as $J\left(E_{control},\;E_{treated}\right)=\frac{\left|E_{control}\;\cap \;E_{treated}\right|}{\;|E_{control}\;\cup \;E_{treated}|}$, where E denotes the set of significant edges (in control and treated). A structural Jaccard index was applied, in which edges connecting the same pair of biomarkers were counted as shared regardless of sign change; sign-changed edges were reported separately. Values range from 0 (no shared edges) to 1 (identical network structure).

Sensitivity analysis was performed using the available summary data to assess the robustness of the network- and distance-based interpretations. First, the correlation-network analysis was repeated using a stricter edge-inclusion threshold (*p* ≤ 0.01) instead of the primary threshold (*p* ≤ 0.05). Network density and Jaccard similarity relative to the corresponding untreated control network were then recalculated under this stricter criterion.

Second, the robustness of the distance-based interpretation was evaluated using a leave-one-biomarker-out procedure. Standardized Euclidean distances from the corresponding untreated control were recalculated after excluding one biomarker at a time from the redox-profile vector. The main distance-based interpretation was considered robust if the relative displacement pattern between HCT-116 and SW-480 cells was preserved after the removal of individual biomarkers.

## 3. Results

### 3.1. Effects of Estradiol on Cell Viability

The effects of different concentrations of estradiol on the viability of the human colorectal cancer cell lines HCT-116 and SW-480 are presented in Table 1. In HCT-116 cells, estradiol induced mild cytotoxicity ranging from 2% to 14% at most tested concentrations. In contrast, treatment with 10^−6^ mol/L estradiol caused a marked decrease in cell viability, resulting in approximately 30% cell death after 24 h. In SW-480 cells, estradiol exerted a proliferative effect, stimulating cell growth by approximately 20–35%, depending on the concentration.

### 3.2. Effects of Estradiol on Cell Migration

**Figure 1:**
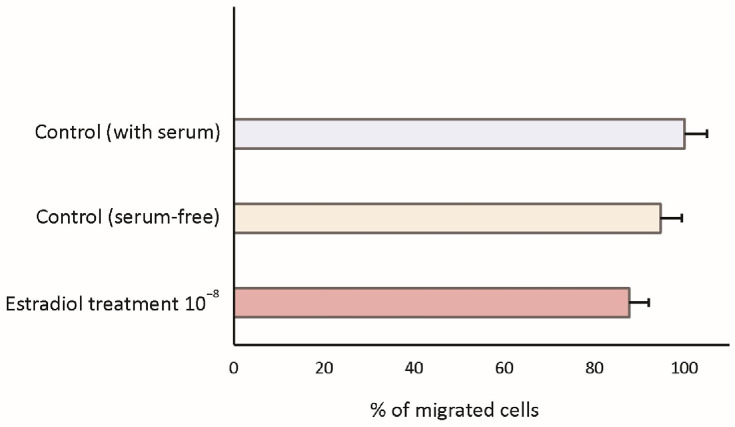
The effect of estradiol at a concentration of 10^−8^ mol/L on the migration of human colorectal cancer cell line HCT-116. The data represent the percentage of migrated cells from three independent experiments, each performed in quadruplicate.

The effect of a viability-neutral concentration of estradiol (10^−8^ mol/L) on the migration of the human colorectal cancer cell line HCT-116 is shown in Figure 1. In the transwell assay, migration ranged from 100% in the positive control with serum to 94.7% in the serum-free negative control, indicating a relatively high level of spontaneous migration in the absence of a chemotactic gradient. Estradiol treatment at a concentration of 10^−8^ mol/L reduced basal cell migration to 87.7% of the positive control, corresponding to an approximate 12% decrease. Notably, estradiol-treated cells migrated less than the serum-free negative control (87.7% vs. 94.7%), although both conditions lacked a chemotactic gradient, indicating a mild inhibitory effect of estradiol on the basal migration of HCT-116 cells under serum-free conditions. Migration was not assessed in SW-480 cells under estradiol treatment, as estradiol-induced proliferation in this cell line could confound the interpretation of transwell migration assay results.

### 3.3. Effects of Estradiol on Redox Biomarker

**Figure 2:**
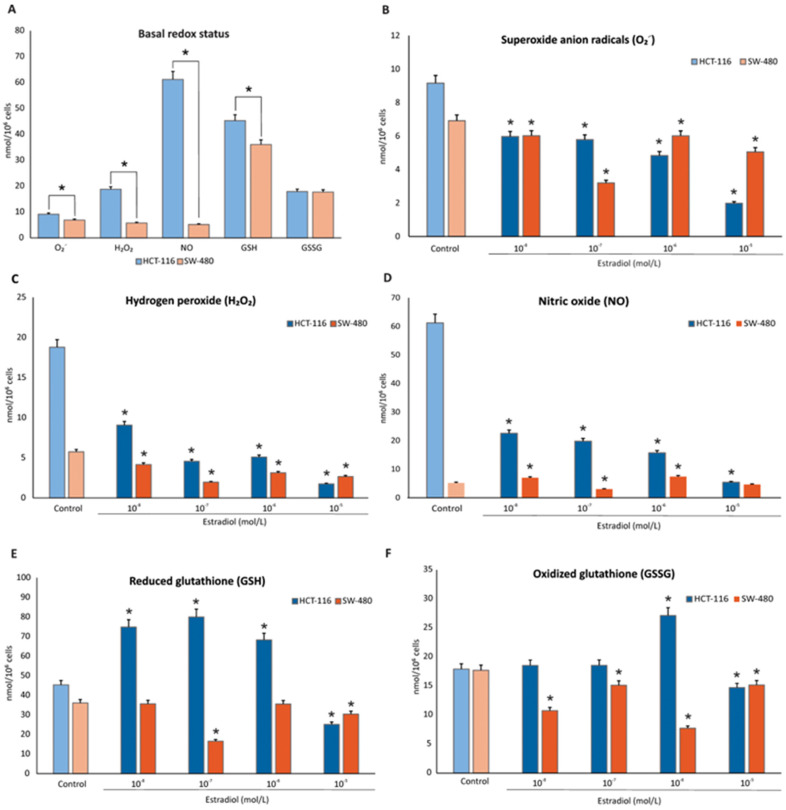
Basal redox status and modulation by different concentrations of estradiol in HCT-116 and SW-480 human colorectal cancer cells. (**A**) Basal redox status of HCT-116 and SW-480 cells; (**B**) superoxide anion radical (O_2_^.−^) levels; (**C**) hydrogen peroxide (H_2_O_2_) levels; (**D**) nitric oxide (NO) levels; (**E**) reduced glutathione (GSH) levels; and (**F**) oxidized glutathione (GSSG) levels. Data are presented as mean ± SEM from three independent experiments, each performed in quadruplicate. * *p* ≤ 0.05 indicates a statistically significant difference between HCT-116 and SW-480 cells in (**A**), and between the control and estradiol-treated groups in (**B**–**F**).

Basal levels (control) of O_2_^.−^, H_2_O_2_, NO, GSH, and GSSG, as well as their modulation by estradiol at different concentrations, are shown in Figure 2 for HCT-116 and SW-480 human colorectal cancer cells. The presented results indicate that, compared with SW-480 cells, HCT-116 cells exhibited approximately 1.3-fold higher O_2_^.−^ levels, about 3-fold higher H_2_O_2_ levels, and nearly 12-fold higher NO levels (Figure 2A).

Estradiol treatment induced a dose-dependent reduction in O_2_^.−^ levels in HCT-116 cells (Figure 2B). In SW-480 cells, estradiol also decreased O_2_^.−^ levels, with the greatest reduction observed at 10^−7^ mol/L (Figure 2B). Estradiol markedly lowered H_2_O_2_ levels in both cell lines, with maximal suppression observed at 10^−5^ mol/L in HCT-116 cells and at 10^−7^ mol/L in SW-480 cells (Figure 2C). A dose-dependent reduction in NO levels following estradiol treatment was observed in HCT-116 cells (Figure 2D). In contrast, in SW-480 cells, estradiol reduced NO levels at 10^−7^ mol/L, whereas treatment with 10^−8^ mol/L and 10^−6^ mol/L resulted in an increase in NO levels (Figure 2D).

Basal GSH levels were approximately 1.25-fold higher in HCT-116 cells than in SW-480 cells, whereas basal GSSG levels were comparable between the two cell lines (Figure 2A). Estradiol treatment increased GSH levels in HCT-116 cells by approximately 1.5-fold to 1.8-fold, except at 10^−5^ mol/L, which resulted in a marked decrease of about 1.8-fold (Figure 2E). In SW-480 cells, estradiol induced a reduction in GSH levels at 10^−7^ mol/L (approximately 2-fold) and 10^−5^ mol/L (approximately 2.5-fold) (Figure 2E). In HCT-116 cells, estradiol treatment modulated GSSG levels, inducing an increase at 10^−6^ mol/L and a decrease at 10^−5^ mol/L (Figure 2F). In SW-480 cells, all applied concentrations of estradiol resulted in reduced GSSG levels, with the most pronounced decrease observed at 10^−6^ mol/L (approximately 2.3-fold) (Figure 2F).

The basal GSH/GSSG ratio, a reliable indicator of cellular redox homeostasis, and its modulation by estradiol at different concentrations are presented in Table 2 for HCT-116 and SW-480 cells. HCT-116 cells exhibited an approximately 1.25-fold higher basal GSH/GSSG ratio compared with SW-480 cells. In HCT-116 cells, estradiol increased the GSH/GSSG ratio at concentrations of 10^−8^ and 10^−7^ mol/L, whereas treatment with 10^−5^ mol/L led to a reduction in this ratio. In SW-480 cells, estradiol increased the GSH/GSSG ratio at 10^−8^ and 10^−6^ mol/L, while a decrease was observed at 10^−7^ mol/L.

### 3.4. Correlations Between Redox Biomarkers

**Figure 3:**
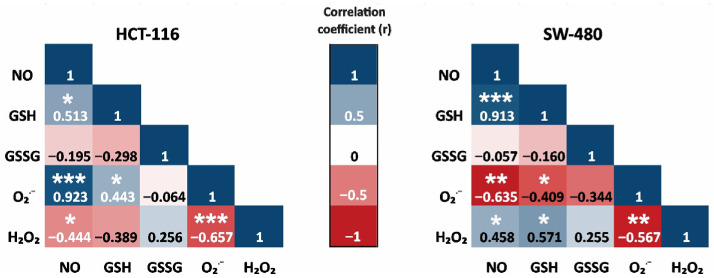
Correlation networks of redox biomarkers (basal redox set-point) in the human colorectal cancer cell lines HCT-116 and SW-480. Data represent Pearson correlation coefficients (r) and the corresponding statistical significance (*p*) for correlations between redox biomarker concentrations. * *p* ≤ 0.05; ** *p* ≤ 0.01; *** *p* ≤ 0.001.

Basal correlations between redox biomarkers in HCT-116 and SW-480 cell lines are presented in Figure 3. In HCT-116 cells, significant positive correlations were observed between O_2_^.−^ and NO, O_2_^.−^ and GSH, and NO and GSH, whereas H_2_O_2_ correlated negatively with both NO and O_2_^.−^. In SW-480 cells, the basal correlation pattern differed from that observed in HCT-116 cells. Positive correlations were found between H_2_O_2_ and NO, H_2_O_2_ and GSH, and NO and GSH, whereas O_2_^.−^ showed negative correlations with H_2_O_2_, NO, and GSH. In both cell lines, correlations between GSSG and the other redox biomarkers were not statistically significant.

**Figure 4:**
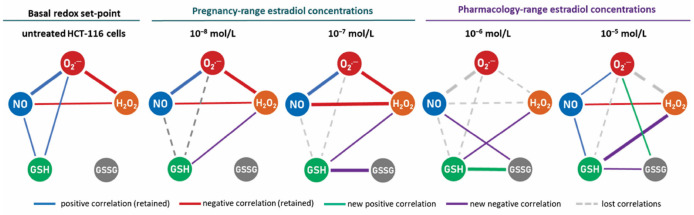
Effects of different concentrations of estradiol on the basal correlation network of redox biomarkers in HCT-116 human colorectal cancer cells. Data represent statistically significant Pearson correlations between redox biomarker concentrations in untreated and estradiol-treated cells.

All basal and estradiol-induced correlations among redox biomarkers are presented in Table S1A,B for HCT-116 and SW-480 cells, respectively, in the Supplementary Materials, whereas only statistically significant correlations are visualized as network graphs. Effects of different estradiol concentration on basal correlations between redox biomarkers in HCT-116 cells are shown in Figure 4. The pregnancy-range estradiol concentrations (10^−8^ and 10^−7^ mol/L) significantly modify the basal correlation network of redox biomarkers in HCT-116 cells. At both concentrations, O_2_^.−^ and NO show a positive correlation. Both O_2_^.−^ and NO also correlate negatively with H_2_O_2_, which in turn is negatively correlated with GSH. At 10^−8^ mol/L estradiol, GSSG is excluded from the correlation network, whereas at 10^−7^ mol/L GSSG shows a negative correlation with GSH. Estradiol at pharmacological concentrations (10^−6^ and 10^−5^ mol/L) exerted a dual effect on the modulation of the basal redox biomarker network in HCT-116 cells. At 10^−6^ mol/L, a positive correlation was observed between GSH and GSSG, whereas H_2_O_2_ showed a negative correlation with GSH, and NO was negatively correlated with GSSG. Under this concentration of estradiol, O_2_^.−^ was disconnected from the correlation network. At 10^−5^ mol/L, both O_2_^.−^ and NO became positively correlated. O_2_^.−^ also positively correlated with GSSG, while NO showed a positive correlation with GSH. Additionally, NO was negatively correlated with H_2_O_2_, which in turn was negatively correlated with GSH, itself negatively correlated with GSSG.

**Figure 5:**
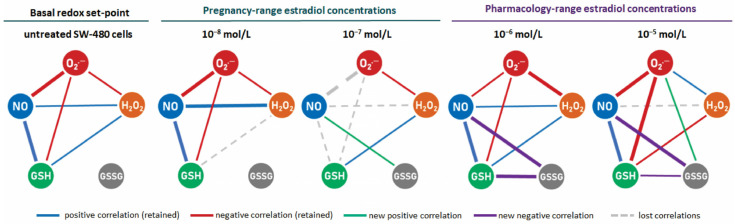
Effects of different concentrations of estradiol on the basal correlation network of redox biomarkers in SW-480 human colorectal cancer cells. Data represent statistically significant Pearson correlations between redox biomarker concentrations in untreated and estradiol-treated cells.

Effects of different estradiol concentrations on basal redox biomarker correlations in SW-480 cells are summarized in Figure 5. Estradiol at pregnancy-range concentrations (10^−8^ and 10^−7^ mol/L) exhibited a dual effect on the basal redox biomarker network. At 10^−8^ mol/L, NO positively correlated with H_2_O_2_ and GSH, while O_2_^.−^ was negatively correlated with H_2_O_2_, NO, and GSH. At this concentration, GSSG was disconnected from the correlation network. At 10^−7^ mol/L, estradiol was associated with a markedly altered basal correlation network, resulting in the formation of two independent subnetworks. One subnetwork was characterized by a positive correlation between NO and GSSG, whereas the second consisted of a negative correlation between O_2_^.−^ and H_2_O_2_ and a positive correlation between H_2_O_2_ and GSH. At pharmacological concentrations (10^−6^ and 10^−5^ mol/L), estradiol in SW-480 cells induced a more integrated and complex network involving all measured redox biomarkers. At 10^−6^ mol/L, H_2_O_2_, NO, and GSH formed a positively correlated cluster, while O_2_^.−^, H_2_O_2_, NO, and GSH formed a negatively correlated cluster. GSSG negatively correlated with NO and GSH. At 10^−5^ mol/L, further alterations in the correlation network were observed: O_2_^.−^ became positively correlated with H_2_O_2_ and GSSG, while NO and GSH maintained a positive correlation. On the other hand, O_2_^.−^ negatively correlated with NO and GSH, and H_2_O_2_ exhibited a negative correlation with GSH, which in turn negatively correlated with GSSG.

### 3.5. Mathematical and Network Analysis of Redox Phenotype Changes

**Figure 6:**
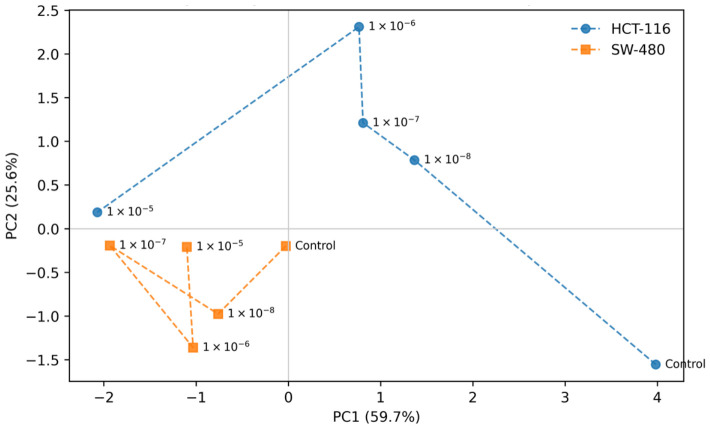
PCA score plot of standardized redox biomarker profiles in HCT-116 and SW-480 colorectal cancer cells under estradiol treatment. Each point represents the mean redox profile (O_2_^.−^, H_2_O_2_, NO, GSH, GSSG) under the indicated condition. PC1 and PC2 together explain 85.3% of total variance. Arrows indicate the direction of estradiol-induced redox phenotype shift from the respective control.

PCA of standardized redox biomarker data (five biomarkers across ten conditions) revealed that PC1 and PC2 together explained 85.3% of total variance (PC1: 59.7%; PC2: 25.6%), indicating that the two-dimensional PC1–PC2 space captures the dominant patterns of redox variation. PC1 was primarily driven by H_2_O_2_, NO, and O_2_^.−^ loadings, reflecting the ROS/NO axis, whereas PC2 was dominated by GSH and GSSG loadings, representing the glutathione redox system. In PCA score space, HCT-116 control was markedly separated from SW-480 control, supporting a distinct basal redox phenotype in each cell line (Figure 6, Table S2 in the Supplementary Materials).

Euclidean distances in the five-dimensional standardized biomarker space demonstrated that estradiol induced progressively larger deviations from the basal redox state in HCT-116 cells (ranging from 3.797 at 10^−8^ mol/L to 6.325 at 10^−5^ mol/L). In contrast SW-480 cells exhibited substantially smaller and non-monotonic deviations (maximum 2.403 at 10^−7^ mol/L), indicating a more stable redox response during 24-h estradiol treatment (Table 3).

**Figure 7:**
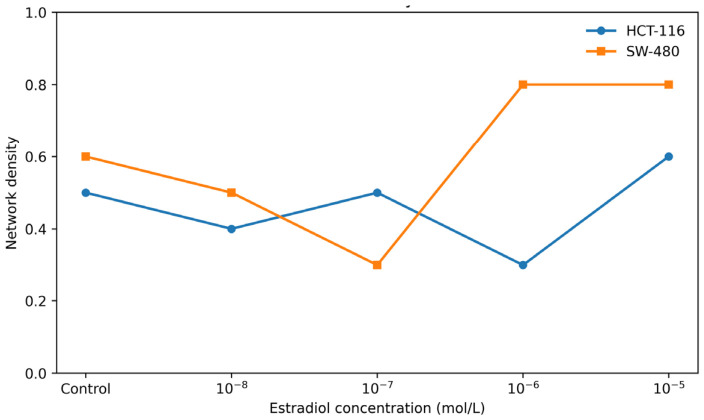
Network density of redox biomarker correlation networks in HCT-116 and SW-480 cells under different concentrations of estradiol. Density is defined as the ratio of statistically significant Pearson correlations (*p* ≤ 0.05) to the maximum number of possible edges (10).

Network analysis revealed that estradiol modulated both the connectivity and the hub structure of biomarker correlation networks in a cell line- and concentration-dependent manner. In HCT-116 cells, the most pronounced network disruption occurred at 10^−6^ mol/L (density D = 0.30; zero retained control edges), with centrality shifting from O_2_^.−^/NO to the GSH/GSSG system (Figure 7). In SW-480 cells, pharmacological concentrations (10^−6^ and 10^−5^ mol/L) led to strongly integrated networks (D = 0.80), with NO and GSH emerging as hub biomarkers (Figure 7, Table S3 in the Supplementary Materials).

**Figure 8:**
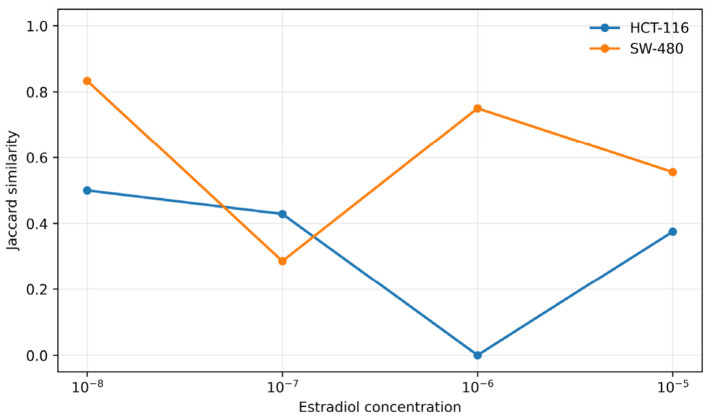
Jaccard similarity index between estradiol-treated and control correlation networks in HCT-116 and SW-480 cells. Values close to 1 indicate structural conservation; values close to 0 indicate substantial structural differences.

The Jaccard index showed that, at 10^−6^ mol/L estradiol, the correlation network in HCT-116 cells differed substantially from the basal state (J = 0.000), with none of the control-state correlations retained. In contrast, SW-480 cells maintained high structural similarity at 10^−8^ mol/L (J = 0.833) and 10^−6^ mol/L (J = 0.750), while the greatest structural divergence was observed at 10^−7^ mol/L (J = 0.286) (Figure 8, Table S4 in the Supplementary Materials).

The sensitivity analysis supported the robustness of the main descriptive mathematical findings. When the correlation networks were reconstructed using the stricter significance threshold (*p* ≤ 0.01), the cell-line-dependent pattern of redox-network alterations was preserved. In HCT-116 cells, the lowest network density was observed at 10^−6^ mol/L (D = 0.10), and the Jaccard similarity with the control network was J = 0.000, indicating that no strongly significant control-state edges were retained. In SW-480 cells, the network remained more connected under pharmacological estradiol treatment, particularly at 10^−6^ mol/L (D = 0.60), with partial preservation of the control network structure (J = 0.429).

The leave-one-biomarker-out distance analysis also confirmed the stability of the distance-based interpretation. After excluding each biomarker separately, HCT-116 cells consistently showed larger standardized Euclidean distances from the corresponding untreated control than SW-480 cells at all matched estradiol concentrations. Therefore, the stronger redox phenotype displacement observed in HCT-116 cells was not driven by a single biomarker alone but reflected a broader redox-profile shift.

## 4. Discussion

This study characterizes the basal redox networks in two human colorectal cancer cell lines (HCT-116 and SW-480) and examines their dynamic modulation by estradiol at both pregnancy-range and pharmacology-range concentrations.

Cancer cells maintain a distinct and adapted redox homeostasis that reflects their proliferative and metastatic potential [41]. Building on this, the present study introduces the concept of a “redox set-point” defined as the characteristic correlation network of ROS and antioxidants that cancer cells maintain to preserve homeostasis and optimize adaptive responses.

According to our results, HCT-116 cells establish a basal redox set-point defined by a tightly interconnected O_2_^.−^-NO-GSH network, supporting both proliferation and migration. In contrast, SW-480 cells display a distinct redox set-point dominated by H_2_O_2_-NO-GSH. Thus, the basal redox set-points of HCT-116 and SW-480 cells differ primarily in the dominant ROS, with O_2_^.−^ predominating in HCT-116 cells and H_2_O_2_ in SW-480 cells.

The high reactivity and short half-life of O_2_^.−^ necessitate rapid conversion into H_2_O_2_ via SOD-mediated dismutation [13,14,42]. H_2_O_2_ acts as a signaling hub that promotes survival and proliferation through PI3K/Akt and MAPK/ERK activation, while suppressing PTEN [43,44,45]. Its ability to traverse membranes via aquaporin 8 (AQP8) enhances autocrine/paracrine communication and metastatic potential [45,46]. Notably, AQP8 expression is higher in HCT-116 than in SW-480 cells [47], indicating efficient H_2_O_2_ export and a redox set-point primarily regulated by intracellular O_2_^.−^. Conversely, SW-480 cells maintain homeostasis through intracellular H_2_O_2_ control. The interplay between O_2_^.−^ and NO further promotes peroxynitrite (ONOO^−^) formation, which depletes both mediators and exerts cytotoxic effects. To preserve signaling and viability, cancer cells activate adaptive defense mechanisms, particularly through the upregulation of GSH [48].

The antioxidant GSH plays a central role in maintaining optimal H_2_O_2_ levels in cancer cells and in attenuating peroxynitrite (ONOO^−^)-mediated cytotoxicity [49]. This occurs both directly, as a reducing agent of ROS, and indirectly, as a cofactor for antioxidative enzymes such as GSH-Px and GST, generating GSSG in the process [42]. Based on the results of this study, basal GSSG levels did not differ significantly between HCT-116 and SW-480 cells. To prevent GSSG accumulation, which can trigger apoptosis signaling pathways [50], cancer cells actively export it via multidrug resistance-associated protein-1 (MRP-1 or ABCC1) [51]. According to the Human Protein Atlas, basal expression of the ABCC1 is relatively low in both HCT-116 cells (nTPM: 2.6) and SW-480 cells (nTPM: 5.1) (https://www.proteinatlas.org/ENSG00000103222-ABCC1/cell+line) (accessed on 3 February 2026) but with comparable levels. This indicates that GSSG efflux capacity is not a major differentiator and does not explain the higher intracellular GSH content and GSH/GSSG ratio observed in HCT-116 cells, pointing instead to differences in GSH biosynthesis or utilization as the key factor in their distinct antioxidative capacity.

Estradiol exerts potent direct and indirect antioxidant effects, thereby critically modulating cellular redox homeostasis. In parallel, it significantly influences signaling pathways involved in cell-cycle regulation and cell survival [22,23,24]. The results of this study demonstrate that estradiol induces dose-dependent alterations in the basal redox homeostasis of HCT-116 and SW-480 cells, which exhibit distinct adaptive responses by modulating their basal redox set-point to preserve viability and proliferative capacity. Estradiol readily crosses cell membranes via passive diffusion. Within the cells, estradiol efficiently scavenges O_2_^.−^ by donating hydrogen from its phenolic hydroxyl group [22], leading to a direct reduction in intracellular O_2_^.−^ levels in both HCT-116 and SW-480 cells. Since H_2_O_2_ is generated through SOD-mediated dismutation of O_2_^.−^ [42], the decrease in O_2_^.−^ consequently results in a concomitant reduction in H_2_O_2_ levels. In parallel, estradiol induces an increase in SOD activity [52] that could further amplify the decline in O_2_^.−^ concentration in examined colorectal cancer cell lines. Additionally, through receptor-dependent mechanisms, estradiol upregulates γ-glutamylcysteine ligase (GCL), the rate-limiting enzyme in GSH biosynthesis [53,54], which can contribute to increasing intracellular GSH levels in HCT-116 cells. Elevated GSH subsequently enhances the GSH-dependent enzymatic detoxification of H_2_O_2_ [42].

In HCT-116 cells, pregnancy-range concentrations of estradiol induce dose-dependent decreases in O_2_^.−^, H_2_O_2_, and NO, accompanied by increases in GSH levels and overall antioxidative capacity (GSH/GSSG). This estradiol-induced increase in antioxidative capacity disrupts the basal redox set-point in HCT-116 cells, shifting it from the optimal toward relatively low O_2_^.−^ and NO levels. This shift in the basal redox set-point is associated with a corresponding reduction in both the migratory and proliferative capacity of HCT-116 cells. Pharmacology-range concentrations of estradiol induced a dose-dependent reduction in O_2_^.−^, H_2_O_2_, and NO levels in HCT-116 cells, while overall antioxidant capacity exhibited a biphasic response. At lower concentrations, estradiol increased both GSH and GSSG levels without altering the basal GSH/GSSG ratio, thereby shifting the redox set-point toward a strongly antioxidative state. This pronounced antioxidative response drives excessive intracellular GSSG accumulation that could overwhelm MRP1-mediated efflux, leading to GSSG retention and induction of apoptosis [50,51]. This process may contribute to the significant cytotoxicity observed in HCT-116 cells. In contrast, higher pharmacology-range concentrations of estradiol markedly reduced the antioxidative capacity of HCT-116 cells, accompanied by depletion of both GSH and GSSG. This was associated with the establishment of a redox set-point defined by a prooxidative-antioxidative network involving low levels of GSH, NO, O_2_^.−^, and GSSG. This loss of the intracellular GSH pool likely represents an adaptive response that limits excessive redox buffering, thereby enabling HCT-116 cells to preserve basal viability under high estradiol exposure.

Overall, HCT-116 cells exhibited greater sensitivity to lower estradiol doses. Both high pregnancy-range and low pharmacological-range estradiol concentrations were associated with reduced proliferation. At the viability-neutral concentration of 10^−8^ mol/L, estradiol was also associated with a mild reduction in basal migration. Although modest, this effect represents an additional functional outcome consistent with the maladaptive response observed in HCT-116 cells and with the broader pattern of progressive redox-profile displacement from the basal redox set-point. However, since only one viability-neutral estradiol concentration was tested and no chemotactic gradient was present under the treatment conditions, this finding should not be interpreted as evidence of altered overall migratory capacity. Furthermore, the high level of spontaneous migration under serum-free conditions may have limited the sensitivity of the assay to detect larger differences.

In contrast to HCT-116 cells, SW-480 cells exhibited a redox adaptive response to all applied estradiol concentrations, manifested by a significant increase in their proliferative capacity, ranging from 20% to 30%. Across both pregnancy-range and pharmacology-range concentrations, estradiol induced a dose-dependent reduction in O_2_^.−^ and H_2_O_2_ levels in SW-480 cells. The response of SW-480 cells to both pregnancy-range and pharmacology-range concentrations of estradiol was biphasic, characterized by distinct modulation of NO and GSH/GSSG levels. At lower pregnancy-range and pharmacology-range concentrations of estradiol, SW-480 cells maintained their basal redox set-point, characterized by decreased H_2_O_2_, elevated NO, and basal-range GSH, accompanied by a decrease in GSSG levels. In contrast, higher pregnancy-range and pharmacology-range concentrations of estradiol induced changes in NO, GSH, and GSSG levels, resulting in the establishment of distinct redox set-points. At higher pregnancy-range concentrations, one redox set-point was dominated by decreased GSH and H_2_O_2_ levels, while the other was characterized by low NO and decreased GSSG levels. At higher pharmacology-range concentrations, one redox set-point was dominated by basal-range NO and low GSH levels, while the other was characterized by low H_2_O_2_, O_2_^.−^, and GSSG levels. Increased iNOS-derived NO supports cancer cell proliferation [18,19,20] by suppressing apoptotic signaling under conditions of redox imbalance [21]. According to literature reports, SW-480 cells exhibit higher basal iNOS expression than HCT-116 cells, which display minimal or undetectable iNOS levels [18,55,56]. Estradiol is known to potently suppress iNOS expression and activity via estrogen receptor–dependent pathways, leading to reduced NO production [57]. Nevertheless, at lower pregnancy-range and pharmacology-range estradiol concentrations, SW-480 cells increased NO production and efficiently eliminated GSSG, thereby enhancing total cellular antioxidative capacity and maintaining the basal redox set-point. Given that estradiol regulates iNOS via receptor-mediated pathways [58], the observed differences in iNOS-derived NO production may reflect the markedly lower ERβ expression in SW-480 cells compared with HCT-116 cells [58]. Owing to reduced ERβ levels, SW-480 cells can efficiently evade the receptor-dependent effects of estradiol, while remaining fully responsive to its direct antioxidative action. Consequently, by circumventing receptor-mediated signaling, SW-480 cells retain the capacity to adapt to the pronounced acute antioxidative challenge posed by estradiol. Given the markedly lower basal iNOS expression in HCT-116 compared with SW-480 cells, estradiol-induced suppression of iNOS signaling may contribute to the pronounced NO depletion and maladaptive redox response in HCT-116 cells, as reflected by their impaired proliferative and migratory capacities. At higher pregnancy-range and pharmacology-range concentrations, SW-480 cells substantially reduced or maintained basal NO levels while concurrently reducing overall antioxidative capacity, in parallel with decreases in O_2_^.−^ and H_2_O_2_ levels.

Overall, SW-480 cells respond to the pronounced antioxidative challenge of estradiol by modulating NO levels and the intracellular GSH/GSSG pool. Through these adaptations, they effectively overcome the strong antioxidative effect imposed by estradiol, which is manifested as enhanced proliferative activity.

Importantly, the classification of the estradiol-induced responses as adaptive or maladaptive was not based on considering individual redox biomarkers as inherently beneficial or detrimental. Rather, it reflected the overall cellular response during the 24-h treatment and the association of changes in the basal redox set-points with opposite functional outcomes. In HCT-116 cells, progressive deviation from the basal redox set-point was associated with inhibition of proliferation and, at the viability-neutral concentration of 10^−8^ mol/L, a mild reduction in basal migration. In contrast, in SW-480 cells, modulation of NO production and the GSH/GSSG pool was associated with enhanced proliferation.

The PCA further corroborates the cell line-specific nature of redox responses. In the PC1–PC2 space, HCT-116 control is markedly separated from SW-480 control, reflecting their distinct basal redox phenotypes dominated by different hub biomarkers. Notably, the PCA trajectory of HCT-116 cells under increasing estradiol concentrations shows a progressive and monotonic departure from the basal state (Euclidean distance 3.8–6.3), indicating a maladaptive cellular response during 24-h estradiol treatment. In contrast, SW-480 cells undergo substantially smaller and non-monotonic deviations (maximum 2.4 at 10^−7^ mol/L), indicating an adaptive cellular response. These findings quantitatively support the concept that distinct basal redox set-points are associated with fundamentally different modes of estradiol responsiveness.

The Jaccard similarity analysis provides complementary evidence for the differential structural plasticity of the two redox networks. The substantial alteration of the basal correlation network in HCT-116 cells at 10^−6^ mol/L (J = 0.000), where no control-state correlation was retained, coincided with the maximal reduction in cell viability, indicating a maladaptive response to 24-h estradiol treatment. Conversely, SW-480 cells maintained high network similarity at 10^−8^ mol/L (J = 0.833) and 10^−6^ mol/L (J = 0.750), with new edges added without losing existing ones at 10^−6^ mol/L, indicating network expansion rather than disruption. This relative preservation of the basal correlation network, combined with the emergence of NO and GSH as hub biomarkers at pharmacological estradiol concentrations, was associated with the pro-proliferative response of SW-480 cells.

In conclusion, this study demonstrates that HCT-116 and SW-480 colorectal carcinoma cells maintain distinct basal redox set-points associated with their divergent malignant phenotypes. HCT-116 cells exhibit a highly reactive O_2_^.−^-centered redox set-point associated with an aggressive proliferative and invasive phenotype, whereas SW-480 cells exhibit a more stable H_2_O_2_-centered redox set-point associated with their comparatively less aggressive phenotype. Estradiol alters these redox set-points in a dose- and cell line-dependent manner, inducing a maladaptive response in HCT-116 cells characterized by inhibition of proliferation and basal migration, associated with a progressive deviation from the basal redox set-point. In contrast, SW-480 cells exhibit an adaptive response characterized by enhanced proliferation associated with modulation of NO production and the GSH/GSSG pool.

These findings support the potential of redox set-point profiling for future personalized therapeutic stratification in colorectal cancer. Characterizing the basal redox set-point of patient-derived tumor cells may help determine whether distinct redox phenotypes are associated with differential estradiol responsiveness. Tumors exhibiting a maladaptive redox response analogous to that observed in HCT-116 cells may, following careful clinical validation, represent candidates for targeted redox modulation using estradiol, whereas tumors resembling SW-480 cells, which exhibit an adaptive response, may be unlikely to benefit from estradiol-based antioxidant approaches. Future studies should extend this approach to a broader panel of redox biomarkers, additional colorectal cancer cell lines, patient-derived tumor tissues, and longer treatment periods to determine whether the identified basal redox set-points and their associations with estradiol responsiveness can be generalized across different experimental models and whether the observed adaptive and maladaptive responses persist over time. Studies combining estradiol with clinically approved colorectal cancer therapeutics are also warranted to evaluate the potential translational relevance of these findings.

## 5. Conclusions

This study provides new insight into the role of redox set-point organization in shaping the response to estradiol in colorectal cancer cells. HCT-116 and SW-480 colorectal cancer cells exhibit distinct basal redox set-points associated with differential responsiveness to estradiol. Estradiol modulates these redox set-points in a dose- and cell line-dependent manner, inducing maladaptive redox imbalance accompanied by suppression of proliferation and basal cell migration in HCT-116 cells, while promoting an adaptive response in SW-480 cells through modulation of NO production and the GSH/GSSG system. These findings identify redox set-point organization as a potential determinant of estradiol responsiveness in colorectal cancer cells and support further exploration of redox-modulating therapeutic strategies, including validation in ex vivo patient-derived tumor models.

## Figures and Tables

**Table 1:** Effects of estradiol at different concentrations on the viability of human colorectal cancer cell lines HCT-116 and SW-480.

		Pregnancy-Range Estradiol		Pharmacology-Range Estradiol	
Estradiol (mol/L)		10^−8^	10^−7^	10^−6^	10^−5^
Cell viability (%)	HCT-116	98.29 ± 4.56	86.94 ± 6.41 *	70.24 ± 5.81 *	93.09 ± 3.60
	SW-480	135.95 ± 15.76 *	128.57 ± 13.61 *	120.15 ± 15.20 *	135.84 ± 20.98 *

Data represent mean ± SEM from three independent experiments, each performed in quadruplicate. * Statistically significant difference in cell viability (*p* ≤ 0.05) compared with untreated cells.

**Table 2:** Effects of estradiol at different concentrations on the GSH/GSSG ratio in human colorectal cancer HCT-116 and SW-480 cells.

Cell Lines	Control	Pregnancy-Range Estradiol (mol/L)		Pharmacology-Range Estradiol (mol/L)	
		10^−8^	10^−7^	10^−6^	10^−5^
HCT-116	2.53	4.03 *	4.31 *	2.51	1.70 *
SW-480	2.01	3.40 *	1.09 *	4.60 *	2.00

Data represent the GSH/GSSG ratio from three independent experiments each performed in quadruplicate. * Statistically significant difference (*p* ≤ 0.05) between the control and estradiol-treated groups.

**Table 3:** Estradiol-induced displacement of the redox profile from the basal redox set-point.

Cell Line	Estradiol	PCA Distance from Control (PC1–PC2)	Standardized Biomarker Distance (5D)
HCT-116	1 × 10^−8^	3.510	3.797
HCT-116	1 × 10^−7^	4.208	4.580
HCT-116	1 × 10^−6^	5.028	5.070
HCT-116	1 × 10^−5^	6.299	6.325
SW-480	1 × 10^−8^	1.069	1.535
SW-480	1 × 10^−7^	1.908	2.403
SW-480	1 × 10^−6^	1.536	2.164
SW-480	1 × 10^−5^	1.073	1.325

## Data Availability

The original contributions presented in this study are included in the article/Supplementary Material.

## Supplementary Materials

The following supporting information can be downloaded at: https://www.mdpi.com/article/10.3390/biomedicines14071577/s1, Table S1: (A) Correlation coefficients between redox biomarkers in untreated (control) and estradiol-treated HCT-116 cells; (B) Correlation coefficients among redox biomarkers in untreated (control) and estradiol-treated SW-480 cells. Table S2: PCA scores by cell line and condition; Table S3: Number of connections and network density; Table S4: Results of Jaccard similarity.
