# Supplementary material for: Estradiol Reshapes Cell-Type-Dependent Basal Redox Set-Points in Colorectal Carcinoma Cells
Source: Biomedicines. 2026 Jul 14;14(7):1577. doi: 10.3390/biomedicines14071577 (PMC13405764; doi:10.3390/biomedicines14071577)
Supplement: Supplementary file 1 [file biomedicines-14-01577-s001.zip › Table S1.pdf]

Table S1. (**A**) Correlation coefficients between redox biomarkers in untreated (control) and estradiol-treated HCT-116 cells (10^{-8}-10^{-5} mol/L).

(**B**). Correlation coefficients among redox biomarkers in untreated (control) and estradiol-treated SW-480 cells (10^{-8}-10^{-5} mol/L).

**(A)**					
**Redox biomarkers**	**$O_2^{\cdot-}$**	**H_2O_2**	**NO**	**GSH**	**GSSG**
	**Untreated (control) HCT-116 cells**				
**$O_2^{\cdot-}$**	-	r = -0.657*** p < 0.001	r = 0.923*** p < 0.001	r = 0.443** p = 0.044	r = -0.064 p = 0.843
**H_2O_2**	r = -0.657*** p < 0.001	-	r = -0.444* P = 0.030	r = - 0.389 p = 0.081	r = 0.256 P = 0.421
**NO**	r = 0.923*** p < 0.001	r = - 0.444* p = 0.030	-	r = 0.513* p = 0.017	r = - 0.195 p = 0.544
**GSH**	r = 0.443 p = 0.044	r = - 0.389 p = 0.081	r = 0.513* p = 0.017	-	r = -0.298 p = 0.436

**GSSG**	r = - .064<br>p = 0.843	r = 0.256<br>p = 0.421	r = -0.195<br>p = 0.544	r = -0.298<br>p = 0.436	-
**HCT-116 cells treated with estradiol at 10^{-8} mol/L**					
**$O_2^{\cdot -}$**	-	r = -0.685***<br>p < 0.001	r = 0.698***<br>p < 0.001	r = 0.081<br>p = 0.705	r = 0.085<br>p = 0.715
**H_2O_2**	r = -0.685***<br>p < 0.001	-	r = -0.579**<br>p = 0.003	r = -0.470*<br>p = 0.020	r = -0.068<br>p = 0.770
**NO**	r = 0.698***<br>p < 0.001	r = -0.579**<br>p = 0.003	-	r = 0.376<br>p = 0.070	r = -0.251<br>p = 0.273
**GSH**	r = 0.081<br>p = 0.705	r = -0.470*<br>p = 0.020	r = 0.376<br>p = 0.070	-	r = 0.035<br>p = 0.882
**GSSG**	r = 0.085<br>p = 0.715	r = -0.068<br>p = 0.770	r = -0.251<br>p = 0.273	r = 0.035<br>p = 0.882	-
**HCT-116 cells treated with estradiol at 10^{-7} mol/L**					

**$O_2^{\cdot -}$**	-	r = -0.617** p = 0.001	r = 0.702*** p < 0.001	r = -0.097 p = 0.675	r = 0.261 p = 0.218
**H_2O_2**	r = -0.617** p = 0.001	-	r = -0.655** p = 0.001	r = -0.489* p = 0.025	r = -0.094 p = 0.664
**NO**	r = 0.702*** p < 0.001	r = -0.655** p = 0.001	-	r = -0.111 p = 0.632	r = 0.261 p = 0.217
**GSH**	r = -.097 p = 0.675	r = -0.489* p = 0.025	r = -0.111 p = 0.632	-	r = -0.812*** p < 0.001
**GSSG**	r = 0.261 p = 0.218	r = -0.094 p = 0.664	r = 0.261 p = 0.217	r = -0.812*** p < 0.001	-
**HCT-116 cells treated with estradiol at 10^{-6} mol/L**					
**$O_2^{\cdot -}$**	-	r = -0.247 p = 0.244	r = 0.308 p = 0.144	r = 0.338 p = 0.106	r = 0.019 p = 0.928
**H_2O_2**	r = -0.247 p = 0.244	-	r = -0.038 p = 0.860	r = -0.505* p = 0.012	r = -0.203 p = 0.340

**NO**	r = 0.308<br>p = 0.144	r = -0.038<br>p = 0.860	-	r = 0.016<br>p = 0.939	r = -0.435*<br>p = 0.034
**GSH**	r = 0.338<br>p = 0.106	r = -0.505*<br>p = 0.012	r = 0.016<br>p = 0.939	-	r = 0.674***<br>p < 0.001
**GSSG**	r = 0.019<br>p = 0.928	r = -0.203<br>p = 0.340	r = -0.435*<br>p = 0.034	r = 0.674***<br>p < 0.001	-
**HCT-116 cells treated with estradiol at 10^{-5} mol/L**					
**$O_2^{\cdot-}$**	-	r = 0.002<br>p = 0.992	r = 0.470*<br>p = 0.021	r = -0.081<br>p = 0.708	r = 0.441*<br>p = 0.031
**H_2O_2**	r = 0.002<br>p = 0.992	-	r = -0.555**<br>p = 0.005	r = -0.781***<br>p < 0.001	r = 0.300<br>p = 0.154
**NO**	r = 0.470*<br>p = 0.021	r = -0.555**<br>p = 0.005	-	r = 0.557**<br>p = 0.005	r = 0.020<br>p = 0.926
**GSH**	r = -0.081<br>p = 0.708	r = -0.781***<br>p < 0.001	r = 0.557**<br>p = 0.005	-	r = -0.471*<br>p = 0.020

**GSSG**	r = 0.441*<br>p = 0.031	r = 0.300<br>p = 0.154	r = 0.020<br>p = 0.926	r = -0.471*<br>p = 0.020	-
**(B)**					
**Redox biomarkers**	**$O_2^{.-}$**	**H_2O_2**	**NO**	**GSH**	**GSSG**
	**Untreated (control) SW-480 cells**				
**$O_2^{.-}$**	-	r = -0.567**<br>p = 0.004	r = -0.635**<br>P = 0.001	r = -0.409*<br>p = 0.047	r = -0.344<br>p = 0.274
**H_2O_2**	r = -0.567**<br>p = 0.004	-	r = 0.458*<br>p = 0.025	r = 0.571*<br>p = 0.004	r = 0.255<br>p = 0.424
**NO**	r = -0.635**<br>p = 0.001	r = 0.458*<br>p = 0.025	-	r = 0.913***<br>p < 0.000	r = -0.057<br>p = 0.861
**GSH**	r = -0.409*<br>p = 0.047	r = 0.571*<br>p = 0.004	r = 0.913***<br>p < 0.000	-	r = -0.160<br>p = 0.619

**GSSG**	r = -0.344<br>p = 0.274	r = 0.255<br>p = 0.424	r = -0.057<br>p = 0.861	r = -0.160<br>p = 0.619	-
**SW-480 cells treated with estradiol at 10^{-8} mol/L**					
**$O_2^{\cdot-}$**	-	r = -0.567**<br>p = 0.004	r = -0.641***<br>p < 0.001	r = -0.462*<br>p = 0.023	r = -0.015<br>p = 0.963
**H_2O_2**	r = -0.567**<br>p = 0.004	-	r = 0.739***<br>p < 0.001	r = 0.396<br>p = 0.056	r = -0.122<br>p = 0.707
**NO**	r = -0.641**<br>p = 0.001	r = 0.739***<br>p < 0.001	-	r = 0.871***<br>p < 0.001	r = -0.108<br>p = 0.738
**GSH**	r = -0.462*<br>p = 0.023	r = 0.396<br>p = 0.056	r = 0.871***<br>p < 0.001	-	r = -0.332<br>p = 0.292
**GSSG**	r = -.015<br>p =.963	r = -0.122<br>p = 0.707	r = -0.108<br>p= 0.738	r = -0.332<br>p = 0.292	-
**SW-480 cells treated with estradiol at 10^{-7} mol/L**					

**$O_2^{\cdot -}$**	-	r = -0.592** p = 0.002	r = -0.300 p = 0.154	r = -0.267 p = 0.208	r = 0.386 p = 0.215
**H_2O_2**	r = -0.592** p = 0.002	-	r = 0.298 p = 0.158	r = 0.446* p = 0.029	r = 0.192 p = 0.549
**NO**	r = -0.300 p = 0.154	r = 0.298 p = 0.158	-	r = 0.371 p = 0.074	r = 0,581* p = 0.047
**GSH**	r = -0.267 p = 0.208	r = 0.446* p = 0.029	r = 0.371 p = 0.074	-	r = 0.349 p = 0.266
**GSSG**	r = 0.386 p = 0.215	r = 0.192 p = 0.549	r = 0.581* p = 0.047	r = 0.349 p = 0.266	-
**SW-480 cells treated with estradiol at 10^{-6} mol/L**					
**$O_2^{\cdot -}$**	-	r = -0.877*** p < 0.001	r = -0.506* p = 0.012	r = -0.433* p = 0.034	r = 0.354 p = 0.259
**H_2O_2**	r = -0.877*** p < 0.001	-	r = 0.516** p = 0.010	r = 0.560** p = 0.004	r = 0.154 p = 0.633

**NO**	r = -0.506* p = 0.012	r = 0.516** p = 0.010	-	r = 0.686** p < 0.001	r = -0.855*** p < 0.001
**GSH**	r = -0.433* p = 0.034	r = 0.560** p = 0.004	r = 0.686*** p < 0.001	-	r = -0.844*** p < 0.001
**GSSG**	r = 0.354 p = 0.259	r = 0.154 p = 0.633	r = -0.855*** p < 0.001	r = -0.844*** p < 0.001	-
**SW-480 cells treated with estradiol at 10^{-5} mol/L**					
**$O_2^{\cdot-}$**	-	r = 0.436* p = 0.033	r = -0.635** p = 0.001	r = -0.766*** p < 0.001	r = 0.586* p = 0.045
**H_2O_2**	r = 0.436* p = 0.033	-	r = 0.005 p = 0.982	r = -0.474* p = 0.019	r = 0.178 p = 0.580
**NO**	r = -0.635** p = 0.001	r = 0.005 p = 0.982	-	r = 0.774*** p < 0.001	r = -0.912*** p < 0.001
**GSH**	r = -0.766*** p < 0.001	r = -0.474* p = 0.019	r = 0.774*** p < 0.001	-	r = -0.737** p = 0.006

**GSSG**	r = 0.586*<br>p = 0.045	r = 0.178<br>p = 0.580	r = -0.912***<br>p < 0.001	r = -0.737**<br>p = 0.006	-

The data represent the Pearson correlation coefficient (r) and the corresponding statistical significance (p) for the correlation between redox biomarkers concentrations. *p ≤ 0.05; **p ≤ 0.01.
