# Supplementary material for: Estradiol Reshapes Cell-Type-Dependent Basal Redox Set-Points in Colorectal Carcinoma Cells
Source: Biomedicines. 2026 Jul 14;14(7):1577. doi: 10.3390/biomedicines14071577 (PMC13405764; doi:10.3390/biomedicines14071577)
Supplement: Supplementary file 1 [file biomedicines-14-01577-s001.zip › Table S2.pdf]

Table S2. PCA scores by cell line and condition

Cell line	Condition	PC1	PC2
**HCT-116**	Control	3.983	-1.554
**HCT-116**	1e-8	1.367	0.785
**HCT-116**	1e-7	0.810	1.211
**HCT-116**	1e-6	0.766	2.310
**HCT-116**	1e-5	-2.071	0.187
**SW-480**	Control	-0.027	-0.199
**SW-480**	1e-8	-0.761	-0.976
**SW-480**	1e-7	-1.935	-0.193
**SW-480**	1e-6	-1.032	-1.361
**SW-480**	1e-5	-1.100	-0.209
