# Supplementary material for: Estradiol Reshapes Cell-Type-Dependent Basal Redox Set-Points in Colorectal Carcinoma Cells
Source: Biomedicines. 2026 Jul 14;14(7):1577. doi: 10.3390/biomedicines14071577 (PMC13405764; doi:10.3390/biomedicines14071577)
Supplement: Supplementary file 1 [file biomedicines-14-01577-s001.zip › Table S3.pdf]

Table S3. Number of connections and network density

Cell line	Condition	Edges	Density	Positive edges	Negative edges
**HCT-116**	$Control$	5	0.5	3	2
**HCT-116**	10^{-8}	4	0.4	1	3
**HCT-116**	10^{-7}	5	0.5	1	4
**HCT-116**	10^{-6}	3	0.3	1	2
**HCT-116**	10^{-5}	6	0.6	3	3
**SW-480**	$Control$	6	0.6	3	3
**SW-480**	10^{-8}	5	0.5	2	3
**SW-480**	10^{-7}	3	0.3	2	1
**SW-480**	10^{-6}	8	0.8	3	5
**SW-480**	10^{-5}	8	0.8	3	5
