# Supplementary material for: Estradiol Reshapes Cell-Type-Dependent Basal Redox Set-Points in Colorectal Carcinoma Cells
Source: Biomedicines. 2026 Jul 14;14(7):1577. doi: 10.3390/biomedicines14071577 (PMC13405764; doi:10.3390/biomedicines14071577)
Supplement: Supplementary file 1 [file biomedicines-14-01577-s001.zip › Table S4.pdf]

Table S4. Results of Jaccard similarity

Cell line	Condition	Control edges	Treated edges	Retained edges	Sign-changed shared edges	New edges	Lost edges	Intersection size	Union size	Jaccard similarity
**HCT-116**	10^{-8}	5	4	3	0	1	2	3	6	0.500
**HCT-116**	10^{-7}	5	5	3	0	2	2	3	7	0.429
**HCT-116**	10^{-6}	5	3	0	0	3	5	0	8	0.000
**HCT-116**	10^{-5}	5	6	3	0	3	2	3	8	0.375
**SW-480**	10^{-8}	6	5	5	0	0	1	5	6	0.833
**SW-480**	10^{-7}	6	3	2	0	1	4	2	7	0.286
**SW-480**	10^{-6}	6	8	6	0	2	0	6	8	0.750
**SW-480**	10^{-5}	6	8	3	2	3	1	5	9	0.556
